# Age-related differences in eye blink-related neural activity and functional connectivity during driving

**DOI:** 10.1016/j.heliyon.2024.e41164

**Published:** 2024-12-14

**Authors:** Emad Alyan, Stefan Arnau, Stephan Getzmann, Julian Elias Reiser, Melanie Karthaus, Edmund Wascher

**Affiliations:** Department of Ergonomics, Leibniz Research Centre for Working Environment and Human Factors, 44139, Dortmund, Germany

**Keywords:** Driving, Aging, Blink-related EEG, Functional connectivity, Eye behavior

## Abstract

Driving is a complex task that requires effective neural processing and coordination, which degrade with aging. Previous studies suggest that age-related changes in cognitive and motor functions can influence driving performance. Herein, we investigated age-related differences and differences between reactive and proactive driving in blink behavior-related potentials, and source-level functional connectivity. Seventy-six subjects participated in two experiments with reactive (19 young, 28 older) and proactive (16 young, 13 older) driving scenarios, consisting of a lane-keeping task with either varying levels of crosswind or curve road, respectively. While blink rate analysis revealed no significant age or driving condition effects, blink duration was notably longer in younger participants. Also, significant age effects were observed in blink-related potentials, mainly in the frontal N2 and occipital P0 and P2 components, with higher amplitudes in younger participants, signifying more efficient neural processing. The parietal N2 component showed significant age and interaction effects, with older individuals showing higher amplitudes in reactive conditions, potentially due to increased cognitive effort and attentional demands. Furthermore, functional connectivity analysis revealed that aging most significantly affects the visual network in the beta band. More specifically, younger participants showed an increase in the clustering coefficient and degrees of the networks, reflecting more robust neural network integration. This pattern of higher connectivity measures in younger participants was also observed in the default mode, control, and limbic networks. Conversely, the dorsal attention network in the theta band showed an increased degree and clustering coefficient in older adults, which could indicate a compensatory mechanism for maintaining cognitive demands. This study highlights the impact of aging on neural activity and connectivity characteristics during driving and emphasizes the requirement of age-tailored interventions, aimed to improve driving safety.

## 1 Introduction

Car driving is a complicated task that requires continuous attention, fast decision-making, and coordination of cognitive processes, such as executive functions, attention, spatial skills and perception, and motor action. Driving is also risky, it remains a leading cause of unintentional injuries and death [1,2]. With increasing age, age-related changes in cognitive abilities (mainly affecting the so-called executive functions) make driving more cognitively demanding for older individuals [3,4]. Although to varying degrees, compared to younger drivers, older drivers may have slower reaction time and weaker cognitive control, making them more prone to distraction; however, they also tend to have more driving experience [[5], [6], [7]].

Besides cognitive decline, age-related changes in sensory and motor function further complicate driving in older adults. According to the study conducted by Ebnali et al. [8], older drivers have more difficulty in maintaining attention and cognitive control to the driving tasks on the one hand, and secondary stimuli on the other hand. Such difficulties may also arise from reduced visual acuity and motor coordination, which are crucial for safe driving [9,10]. Consequently, older drivers seem to increase their mental effort to compensate for neurocognitive decline and allocate more cognitive resources to respond adequately to critical traffic situations [11,12]. Electroencephalography (EEG) might help to identify the origin and amount of altered ability, as many studies have employed EEG for measuring cognitive load and investigating neural mechanisms underlying driving performance. For instance, Getzmann et al. [13] found that older drivers tend to exert more mental effort to maintain driving performance, which was reflected in increased EEG theta power and subjective fatigue. Moreover, the accumulation rates of fatigue derived from the EEG signal differ with age, with older drivers showing a quicker onset of fatigue compared to their younger counterparts [14].

Eye blinks were traditionally seen as artifacts in the EEG data stream. However, since blinks are a reoccurring and natural behavior, they may also serve as natural time-locking events, allowing for event-related analysis of cognitive processing in naturalistic environments [15]. The results of a study by Wascher et al. [15] support the idea of eye-blink-related potentials (bERPs) being meaningful markers for cognitive processes. The blink-evoked fronto-central N2 and theta activity during cognitive tasks increased compared to at rest and physical tasks, indicating that cognitive control was boosted, whereas the continuous nature of physical tasks was confirmed by the posterior P3 reduction. Another study by Wascher et al. [16] investigated the impact of visual demands on blink-related EEG activity during walking, showing that blink-related measures were able to reflect cognitive load during naturalistic activities [16]. Findings by Alyan et al. [17] on blink-related EEG changes in a steam-engine operating task showed a similar pattern, such that active task conditions implied more cognitive engagement, as reflected by changes in N1 and P3 amplitudes. Similar results were also found in another study showing that proactive compared to reactive driving tasks increased cognitive load as reflected in occipital N1, frontal N2, parietal P3, and occipital P2 amplitudes [18]. Additionally, a very early positive bERP component occurs at the peak of the blink signal (during eye reopening) that has been observed in our previous studies [[16], [17], [18]]. We refer to this component as P0, which appears in the posterior cluster, particularly the occipital region, and it exhibits variations with task changes. However, this component has not been thoroughly investigated, and its potential impact could be further explored in this research. Therefore, we argue that considering eye blinks as automatic segmentations of continuous EEG activity bears a high potential to infer changes in cognitive load during various tasks. However, despite these studies on blink-related EEG measures and their link to cognitive processing, no studies have specifically examined age differences in these measures.

The present study intends to address this scientific blind spot as well as to analyze how eye blink-related functional connectivity (bFC) varies with age under diverse driving conditions. Prior research found that, during high load demands, theta connectivity between brain regions is elevated, and the changes in connectivity happen over the post-eye blink activity [17]. Connectivity analysis can also help understand how the functional connections within the brain differ between younger and older individuals in response to different driving conditions. Previous research has indicated that functional networks in the brain change with age, influencing cognitive performance [[19], [20], [21], [22]]. Age-related differences in brain function have been observed, with older adults showing altered functional connectivity at rest, particularly a decrease in within-network connectivity and an increase in between-network connectivity [19]. Graph theory analyses have further clarified the concepts of modularity and clustering in relation to the observed changes in brain aging. Reduced modularity and clustering indicate a decrease in the brain's inherent community structure and the network properties of related nodes. Knyazev et al. [21] confirmed the above alterations by presenting that modularity and clustering were diminished within the networks of both beta and gamma bands in the older group, highlighting the shift towards a more random network organization in the older group [21]. In their turn, Javaid et al. [23] expound that network topology features were reduced among the elderly in a visual working memory task, and the elderly were found to have lower local and global efficiency and clustering coefficients in comparison to other age groups [23].

The current study aims to determine age differences in bERPs and bFC during reactive and proactive driving. While both markers provide insights into brain activity surrounding blinks, they differ in their nature and the information they offer. bERPs, similar to traditional event-related potentials, are characterized by time-locked changes in brain electrical activity following a specific event, in this case, an eye blink. They offer insights into the temporal dynamics of blink-related processing, revealing how the brain responds to and processes these events over time. On the other hand, bFC examines the statistical dependencies between brain regions following blinks, revealing how different areas of the brain interact and synchronize their activity in relation to these events. Therefore, the bERPs will allow us to understand how aging affects neural responses to blinks and the cognitive demands of different driving tasks. The bFC analysis will support the identification of age-related changes in the functionality of different brain connections and, consequently, help explain the neural mechanisms of cognitive decline. Two different driving scenarios were employed, a lane-keeping task on a winding road (proactive driving) and with changing crosswind (reactive driving), and the EEG of young and older drivers during driving was recorded. While some data from these experiments have been previously analyzed [18,24,25], the present analyses uniquely focus on understanding age differences in blink-related EEG activity and connectivity, which were not covered in those previous works. These contributions provide insights into how aging affects the neural efficiency and connectivity of blink-related neural responses, which have implications for safe driving interventions in older adults. Assuming that the bERPs in older adults are expected to be different, and their connectivity is expected to be worse than that of younger adults, we hypothesize that driving tasks would impose more cognitive load on the brains of older adults. Consequently, we postulate that such a change would be less pronounced in bERPs during proactive driving, which involves controlled responses, than during reactive driving, which requires immediate responses. The bFC measures might indicate that older adults would have less within-network connectivity, as hinted at by Deery et al. [19], which should be reflected by lower local clustering and degree measures.

## 2 Materials and methods

### 2.1 Subjects

A total of 76 subjects participated in the two different driving experiments, reactive and proactive driving, after providing written informed consent. Forty-seven subjects were recruited for the reactive driving and twenty-nine for the proactive driving experiments. The reactive driving group had nineteen young subjects (10 females and 9 males; average age 24.6 ± 3.0 years) and twenty-eight older subjects (14 females and 14 males; average age 64.6 ± 3.5 years). The proactive driving group had sixteen young subjects (8 females and 8 males; average age 24.1 ± 3.0 years) and thirteen older subjects (7 females and 6 males; average age 63.1 ± 4.5 years). Part of the data from these experiments is already published under different analyses [18,24,25]. All participants used a car at least twice a week during the last 3 years. The possible participants of the study were screened for eligibility according to the presence of neurologic or psychiatric disorders, substance use affecting the central nervous system, corrected or normal vision, and hearing, before their admission into the study. Each participant received up to 30€/hour for taking part in the experiment. The study was approved and performed following the Declaration of Helsinki by the local ethics committee at the Leibniz Research Centre for Working Environment and Human Factors (IRB approval number: 75–2014).

### 2.2 Experimental procedure

**Fig. 1:**
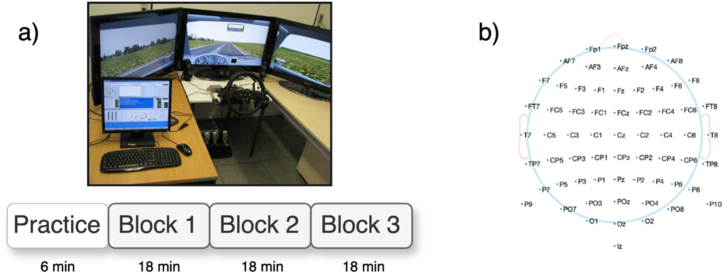
(a) Experimental setup with an initial practice block and three subsequent blocks, each consisting of nine triplets. (b) The EEG cap layout for 64 electrodes.

The two driving scenarios were carried out in a stationary driving simulator (ST Sim; ST Software B.V., Groningen, Netherlands) and explained in detail in Refs. [24,25]. In the reactive driving scenario, the subjects drove on a straight, two-lane road in a reactive driving scenario with instructions to keep their vehicle within the lane. They dealt with a variable crosswind effect created from a complex pattern of eight superimposed sine waves, phase-shifted to simulate the randomness of crosswind influences. This scenario required participants to react immediately to unpredictable changes in crosswind, which could lead to higher cognitive demand compared to proactive driving. The unpredictable nature of crosswind effects meant that participants had to allocate more cognitive resources to attentional control and motor coordination, thereby increasing the workload associated with the task. In the proactive condition, the drivers followed a one-way road with variably curved paths, which required them to plan ahead and maintain control while navigating curves. Although proactive driving involved less immediate responses compared to reactive driving, it still required sustained focus and careful vehicle control over an extended period, engaging both executive functions and spatial skills. The two scenarios were set up against a monotonous grassland background with no visual distractions, and the driving speed was maintained at 50 km/h. At the beginning of the experiment, there was a practice block lasting 6 min, followed by three experimental blocks; the 54 min of uninterrupted testing were divided randomly into three blocks (see Fig. 1a).

### 2.3 Data acquisition

EEG data were recorded using the Biosemi ActiveTwo system (BioSemi, NL) with 64 scalp electrodes positioned according to the International 10–10 system. The scalp-electrode layout is shown in Fig. 1b. Two more electrodes were placed on the mastoids (L/R). The system operates in accordance with the CMS/DRL principle for common mode noise detection using a two-wire active electrode setup. Data were sampled at a rate of 2048 Hz, with a bandwidth of DC to 140 Hz. The impedance of the electrode was kept below 10 kΩ throughout the recording.

### 2.4 Data processing

Data were preprocessed and analyzed through custom MATLAB scripts and the EEGLAB toolbox [26]. The data were initially preprocessed by means of finite impulse response (FIR) filtering using the eegfiltnew function, which applied a high-pass filter at 0.1 Hz and low-pass at 40 Hz to decrease potential environmental and muscular artifacts. The data were then downsampled at 256 Hz. Subsequently, the clean_artifacts function was used to identify and label any bad channels, defined as having a correlation less than 0.80 with neighboring channels and a maximum bad time fraction of 0.3. Following this, the data were re-referenced to a common average. Next, we applied a high-pass zero-phase Hamming-window FIR filter with a cutoff frequency of 1.5 Hz. The filtered data were decomposed into statistically independent components (ICs) using AMICA [27]. These ICs were projected onto the average referenced data.

Given the focus of the study on eye blink-related EEG data, the blinking activities were extracted from the decomposed ICs. Several eye-related ICs were found and eye-blink-related ICs representing eye blinks were separated from the saccade-related ICs by calculating the Pearson correlation coefficient between the left and right anterior channels [18]. Blink detection is accomplished through the EEG BLINKER algorithm [28], with the detected blink peaks marked as event markers in the “EEG. structure” for subsequent EEG data segmentation. The blink properties, such as blink rate and duration, were extracted. In this context, the blink rate represents the number of blinks per minute, while the blink duration indicates the length of each blink in seconds. The duration was calculated as the difference between the right base and left base of the blink event.

Following the extraction of blink properties, we applied IC apportioning using the IClabel algorithm to retain only brain-related ICs. This algorithm classifies ICs based on their probability of representing various sources, including brain activity, muscle activity, eye movements, heartbeats, line noise, channel noise, and other artifacts. Components ruled as less than 30 % brain or greater than 30 % eye, muscle, heart, channel, or other non-brain were discarded. Intervals from −500 ms to 1000 ms around the eye blink peak were epoched. The epochs were cleaned using the pop_autorej function from EEGLAB to automatize the rejecting of epochs with fluctuations larger than an absolute threshold of 500 μV and a standard deviation threshold of 5, iterating repeatedly, up to rejecting 10 % of the epochs per iteration. The average remaining epochs per subject for both driving scenarios was 780, with a minimum of 279. To ensure an equal number across subjects, these epochs were randomly downsampled to the minimum.

### 2.5 Blink-related potentials

The event-related potentials (ERP) were time-locked to the peak of eye blinks for a comprehensive blink-related ERP analysis. The segmented data underwent baseline correction using the interval ranging from −300 to −100 ms relative to the maximum of the blink. Amplitude analyses for different ERP components were conducted at various electrode sites: frontal (F1, F2, Fz, FC1, and FC2), parietal (Pz), and occipital (PO3, POz, and PO4) to evaluate the effect of experimental factors on the different ERP components. A grand average waveform for each driving condition (proactive and reactive) was created by averaging the ERP waveforms across all subjects (both age groups combined). This methodology helped ensure that the majority of the brain response to blinks was captured, decreasing the variation between participants and within a given participant across time. Peak latencies for the peaks (positive components) and troughs (negative components) within certain predefined latency windows were measured on grand average waveforms to quantify the ERP components of interest. The computed latencies for the reactive condition are as follows: frontal (N2:261.72 ms), parietal (P2:167.97 ms, N2:234.38 ms, and P3:308.59 ms), and occipital (P0: −3.91 ms, N1:109.38 ms, and P2:214.84 ms). The P0 component represents the fast positive component immediately while the eye re-opening. For the proactive condition, the latencies were: frontal (N2: 261.72 ms), parietal (P2: 164.06 ms, N2:218.75 ms, P3:300.78 ms), and occipital (P0: −3.91 ms, N1:117.19 ms, and P2:238.28 ms). Data for each subject were then averaged over a ±15 ms window surrounding these peaks.

### 2.6 Functional connectivity

The functional connectivity analysis was conducted using the FieldTrip toolbox [29]. To evaluate functional connectivity within the 0–1000 ms post-eye blink data, we utilized amplitude envelope correlation (AEC) on source-level data. This approach could effectively address volume conduction issues [30]. The AEC was selected due to its stability and reliability [31]. Our method, adapted from Ref. [32], employed cortical source localization using the Schaefer atlas with 100 regions, covering seven networks: Visual (Vis), Somato-Motor (SM), Dorsal Attention (DA), Salience-Ventral Attention (SVA), Limbic, Control (CON), and Default mode network (DMN). Electrophysiological activity was estimated using a linearly constrained minimum variance (LCMV) beamformer [33]. The lead fields were constructed from a Montreal Neurological Institute (MNI) template, and spatial filters were generated from covariance matrices with 5 % regularization. The dipole orientation is fixed to align with the direction of maximum variance.

**Fig. 2:**
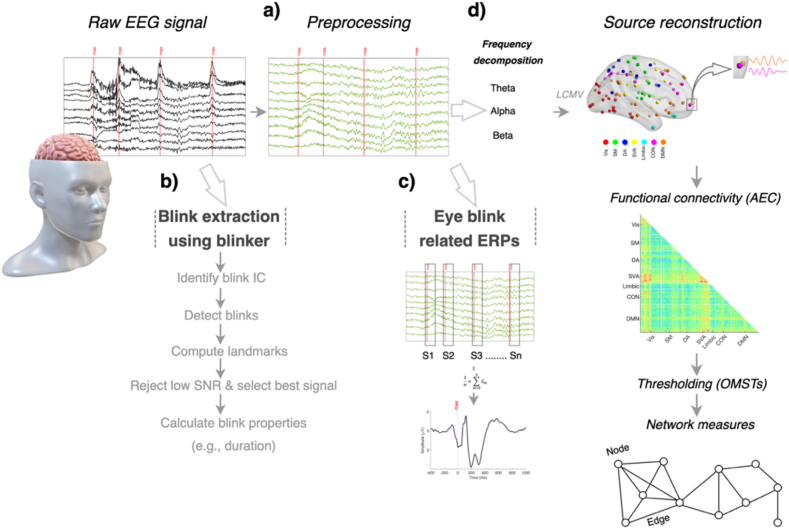
Schematic representation of the analytical framework involved in data processing. EEG data were recorded during a driving simulator task involving proactive and reactive conditions. (a) Data preprocessing included filtering, as well as artifact and component rejection, to obtain clean EEG signals. (b) Blink detection was performed by isolating eye-blink components using Independent Component Analysis and extracting blink properties. (c) Blink-related potentials were computed by time-locking EEG signals to blink events, followed by age- and condition-specific ERP analysis. (d) Functional connectivity was analyzed at the source level, with data projected from sensor space using the LCMV beamformer. The AEC was used to generate a high-resolution connectivity matrix, later binarized via OMSTs. Finally, network measures, including clustering coefficient and degree, were extracted for graph-based analysis.

For each frequency band, including theta (4–8 Hz), alpha (8–13 Hz) and beta (13–30 Hz), the Hilbert transform was applied to compute analytical signals. This provided the amplitude envelope of the source time series. Subsequently, Pearson correlations between orthogonalized amplitude envelopes were used to create a 100 × 100 connectivity matrix for each epoch. These matrices were then averaged across epochs to obtain final connectivity matrices for each frequency band. The connectivity matrix for each subject was then filtered and binarized using the orthogonal minimal spanning trees (OMSTs) method [34]. This data-driven approach addresses the limitations of proportional thresholding by including both weak and strong connections, optimizing global cost efficiency, and providing a more detailed representation of underlying neuronal networks. BrainNet Viewer was employed to visualize the functional connectivity [35]. Additionally, two graph measures were calculated for extracted networks, the clustering coefficient and degree. The former measures how close the neighbors of a node are to forming a clique, indicating local network density. The latter estimates the number of links connected to each node, reflecting its centrality within the network. Both measures, which are implemented in the MATLAB Brain Connectivity Toolbox [36], were used for our network analyses. The main methodological steps of the data processing performed in the present work are summarized in Fig. 2.

### 2.7 Statistical analysis

We conducted two-way ANOVAs with Age (old, young) and Driving Condition (reactive, proactive) as factors on ERP components, eye blink behavior (blink rate and blink duration), and connectivity measures (clustering coefficient and degree) at α = 0.05, using the anovan function in MATLAB. Post-hoc analyses were performed with the Tukey-Kramer method to correct for multiple comparisons, utilizing the multcompare function in Matlab. We tested this separately for the main effects and for the interactions, for all ERP components. The eye blink behavior metrics were also analyzed to examine the impacts of Age and Driving Conditions. We then used two-way ANOVAs to test the effect of these factors on the connectivity measures for the seven brain networks.

## 3 Results

### 3.1 Blink behavior and age

**Fig. 3:**
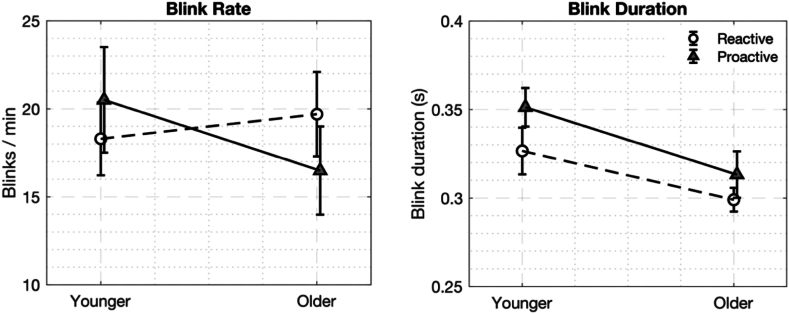
Comparison of blink rate (blinks per minute) and blink duration (in seconds) between younger and older participants under reactive and proactive driving conditions. Error bars represent the standard error of the mean.

The effects of age and driving condition (reactive vs. proactive) on blink rate and blink duration were examined as shown in Fig. 3. For blink rate, a two-way ANOVA revealed no significant main effects of age (F(1,72) = 0.243, p = 0.624, η2 = 0.003) or driving condition (F(1,72) = 0.035, p = 0.852, η2 < 0.001). Additionally, there was no significant interaction between age and driving condition (F(1,72) = 1.039, p = 0.312, η2 = 0.014). In contrast, blink duration demonstrated significant differences, with a main effect of age (F(1,72) = 9.145, p = 0.003, η2 = 0.106), and with younger participants showing longer blink durations compared to older participants. Although the main effect of the driving condition was not significant (F(1,72) = 3.217, p = 0.077, η2 = 0.037), there was a trend towards longer blink durations in the proactive condition. The interaction between age and driving condition was not significant (F(1,72) = 0.233, p = 0.631, η2 = 0.003).

### 3.2 Age effects on blink-related ERPs

**Fig. 4:**
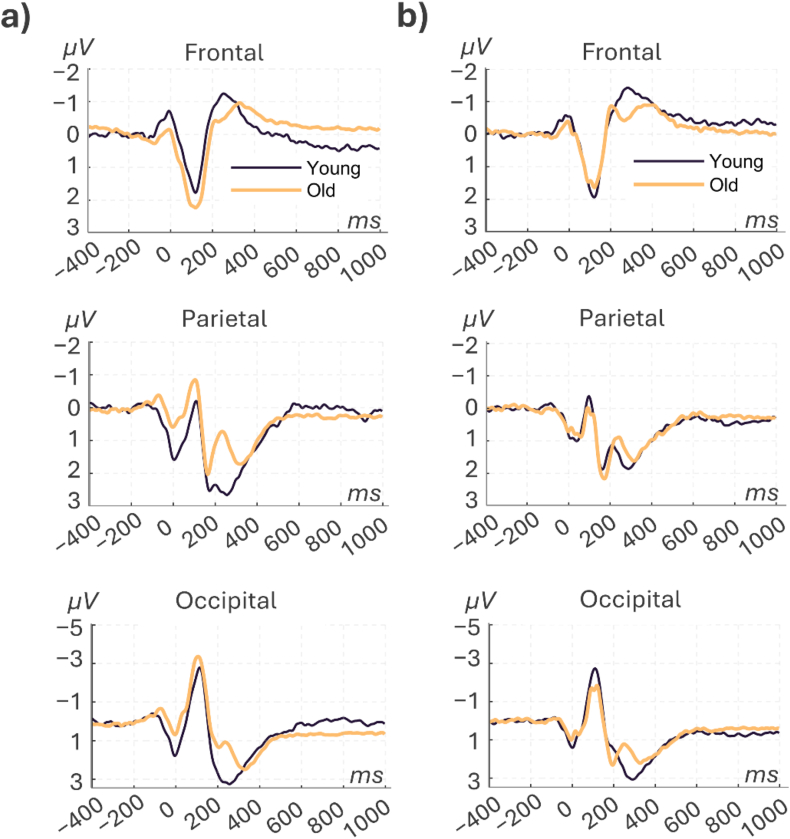
Grand-average eye blink-related potential signals for frontal (F1, F2, Fz, FC2, FC1), parietal (Pz), and occipital (PO3, POz, PO4) brain areas in younger (black) and older adults (gold) during reactive (a) and proactive (b) driving conditions.

**Fig. 5:**
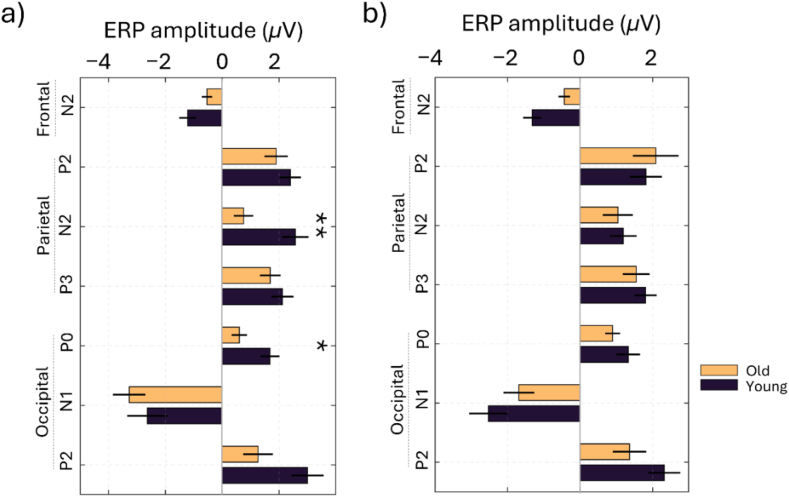
Mean and standard error of bERPs for older and younger adults during reactive (a) and proactive (b) driving conditions. Asterisks indicate post hoc significance, with ∗p < 0.05 and ∗∗p < 0.01.

A two-way ANOVA was also conducted to examine the effects of age, driving condition, and their interaction on the bERP components in the frontal, parietal, and occipital regions (see Table 1 and Fig. 4, Fig. 5). The results for the frontal N2 component revealed a significant main effect of age (F(1, 72) = 10.29, p = 0.002, ƞ^2^ = 0.125), while the main effect of driving condition and the interaction between age and driving condition was not significant (F(1, 72) = 0.0002, p = 0.99, ƞ^2^ < 0.01). However, post-hoc comparisons showed that the differences between old and young participants under reactive and proactive driving conditions were not significant (reactive, mean difference (MD) = 0.680, p = 0.122; proactive, MD = 0.883, p = 0.104). For the parietal region, the ANOVA results of the P2 and P3 components indicated no significant main effects or interaction (see Table 1). The parietal N2 component showed a significant main effect of age (F(1, 72) = 5.51, p = 0.022, ƞ^2^ = 0.065) and a significant interaction between age and driving condition (F(1, 72) = 3.98, p = 0.049, ƞ^2^ = 0.047), but no main effect of driving condition (F(1, 72) = 1.71, p = 0.196, ƞ^2^ = 0.020). Post-hoc analysis revealed a significant difference between older and younger participants under reactive driving conditions, with higher N2 amplitude observed for older participants (MD = −1.821, p = 0.005). There were no significant differences in other comparisons (p > 0.05).

**Table 1:** Analysis of bERP components by brain region, age, and driving condition using two-way ANOVA.

Brain region	ERP	Source	Mean squares	F(1,72)	p-value	ƞ^2^	Post-hoc: Old vs. Young		
							Driving conditions	MD	P-value
Frontal	N2	Age	10.73	10.29	0.002	0.125	Reactive	0.68	0.1222
		Driving condition	0	0	0.9895	0	Proactive	0.88	0.1038
		Interaction	0.18	0.17	0.6781	0.002			
Parietal	P2	Age	0.23	0.06	0.8108	0.001	Reactive	−0.5	0.8318
		Driving condition	0.7	0.18	0.6759	0.002	Proactive	0.27	0.9829
		Interaction	2.63	0.66	0.4179	0.009			
	N2	Age	17.02	5.51	0.0217	0.065	Reactive	−1.82	0.005
		Driving condition	5.27	1.71	0.1958	0.02	Proactive	−0.15	0.9959
		Interaction	12.3	3.98	0.0499	0.047			
	P3	Age	2.07	0.76	0.3861	0.01	Reactive	−0.43	0.8157
		Driving condition	0.95	0.35	0.5571	0.005	Proactive	−0.26	0.9758
		Interaction	0.14	0.05	0.8242	0.001			
Occipital	P0	Age	10.06	5.77	0.0189	0.072	Reactive	−1.08	0.037
		Driving condition	0.02	0.01	0.9188	0	Proactive	−0.44	0.8135
		Interaction	1.81	1.04	0.3117	0.013			
	N1	Age	0.17	0.02	0.8794	0	Reactive	−0.64	0.8504
		Driving condition	12.6	1.76	0.1883	0.023	Proactive	0.84	0.8362
		Interaction	9.6	1.34	0.2503	0.018			
	P2	Age	31.91	5.75	0.0191	0.072	Reactive	−1.74	0.07
		Driving condition	1.51	0.27	0.6039	0.003	Proactive	−0.95	0.7001
		Interaction	2.73	0.49	0.4858	0.006

For the occipital electrode cluster, a significant main effect of age was observed (F(1, 72) = 5.77, p = 0.0189, ƞ^2^ = 0.072) for the P0 component. However, the main effect of the driving condition and the interaction were not significant (F(1, 72) < 1.04, p > 0.05, ƞ^2^ < 0.013). Post-hoc comparisons showed a significantly higher P0 amplitude for young participants under reactive driving conditions (MD = −1.08, p = 0.037). Other comparisons did not reveal any significant differences (p > 0.05). The analysis for the occipital N1 component did not show significant main effects or interactions (see Table 1). For the occipital P2 component, there was a significant main effect of age (F(1, 72) = 5.75, p = 0.019, ƞ^2^ = 0.072), while the main effect of driving condition and the interaction between age and driving condition showed no significant results (F(1, 72) < 1, p > 0.05, ƞ^2^ < 0.01). Post-hoc analysis only showed a trend towards significance for the difference between older and younger participants under reactive driving conditions, with higher P2 amplitude observed for younger participants (MD = −1.74, p = 0.07). Other comparisons did not show significant differences (p > 0.05).

### 3.3 Age-related differences in network connectivity

**Fig. 6:**
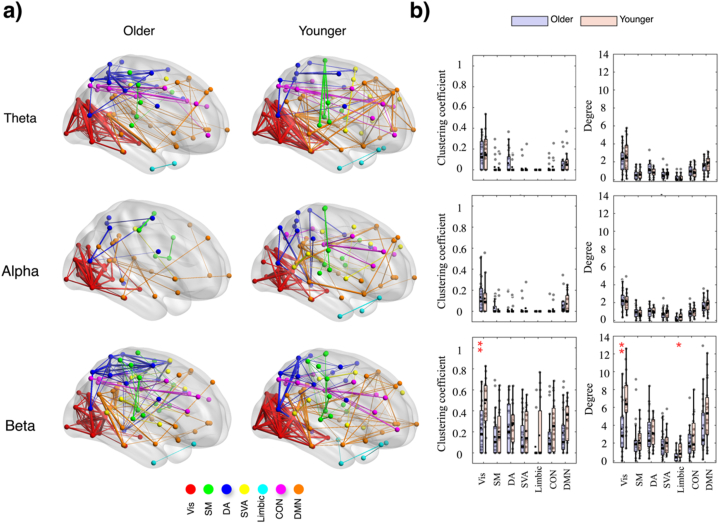
Functional connectivity during reactive driving. a) Brain networks of EEG frequency bands (theta, alpha, and beta) for older and younger adults. Distinct brain networks are coded with different colors: Vis (red), SM (green), DA (blue), SVA (yellow), Limbic (cyan), CON (purple), and DMN (orange). The patterns of connectivity reveal variations in the network structures among the two age groups. b) Bar plots of the clustering coefficient and degree measures for older and younger adults across the seven brain networks and the three EEG frequency bands. Post hoc significance is indicated by asterisks, with ∗p < 0.05 and ∗∗p < 0.01.

**Fig. 7:**
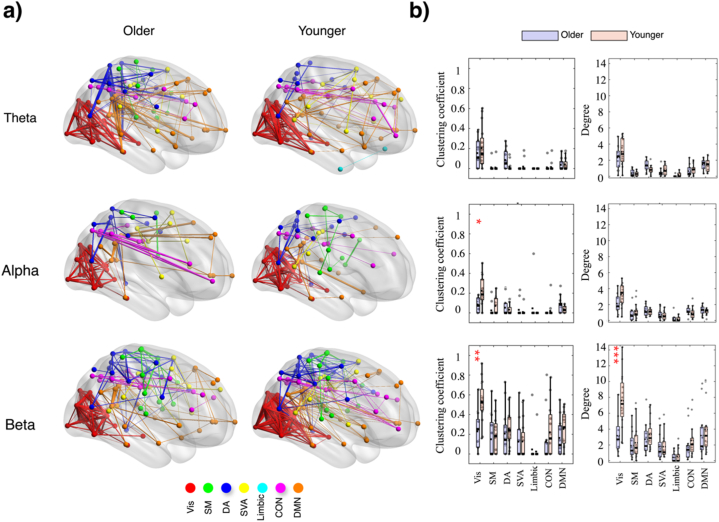
Functional connectivity during proactive driving. a) Brain networks of EEG frequency bands (theta, alpha, and beta) for older and younger adults. Distinct brain networks are coded with different colors: Vis (red), SM (green), DA (blue), SVA (yellow), Limbic (cyan), CON (purple), and DMN (orange). The patterns of connectivity reveal variations in the network structures among the two age groups. b) Bar plots of the clustering coefficient and degree measures for older and younger adults across the seven brain networks and the three EEG frequency bands. Post hoc significance is indicated by asterisks, with ∗p < 0.05, ∗∗p < 0.01 and ∗∗∗p < 0.001.

Fig. 6, Fig. 7 reveal general variations in clustering coefficient and degree measures across seven brain networks for three frequency bands (theta, alpha, beta) during reactive and proactive driving, respectively. Detailed statistics are provided in the supplementary material. For instance, in the beta band, the visual network displayed significant age effects for both clustering coefficient (F(1,72) = 25.50, p < 0.001, η^2^ = 0.257) and degree (F(1,72) = 31.14, p < 0.001, η^2^ = 0.298). However, the post-hoc analysis revealed that younger participants showed significantly higher values for both measures under both reactive (clustering coefficient: MD = −0.234, p = 0.004; degree: MD = −3.217, p = 0.002) and proactive (clustering coefficient: MD = −0.302, p = 0.003; degree: MD = −4.404, p = 0.0006) conditions, indicating higher connectivity in younger participants. The DMN showed significant age effects for the clustering coefficient (F(1,72) = 5.97, p = 0.017, η^2^ = 0.075) and approaching significance for the degree (F(1,72) = 2.84, p = 0.096, η^2^ = 0.037), with younger participants having higher values in both measures. The post-hoc analysis showed no significant pairwise differences between groups (p > 0.05). The control network exhibited significant age effects for clustering coefficient (F(1,72) = 4.03, p = 0.048, η^2^ = 0.052) and the limbic network for degree (F(1,72) = 5.07, p = 0.027, η^2^ = 0.061), with younger participants showing higher connectivity. The post-hoc analysis for degree revealed significant pairwise differences under reactive conditions for the limbic network (MD = −0.644, p = 0.021), while no significant pairwise differences were observed for the clustering coefficient of the control network.

In the theta band, the clustering coefficient showed minimal significant effects across networks. For the DA network, a significant effect of age was observed (F(1,72) = 6.93, p = 0.01, η^2^ = 0.088), with older participants exhibiting higher clustering coefficients. The post-hoc analysis did not reveal significant pairwise differences. Similarly, the degree measure for the DA network showed a significant age effect (F(1,72) = 8.73, p = 0.004, η^2^ = 0.108), with older participants having higher degrees. For the SM network, the degree measure showed a significant effect on driving conditions (F(1,72) = 4.37, p = 0.040, η^2^ = 0.057), indicating that reactive driving resulted in higher degrees. The SVA network also showed a significant effect of age for the degree (F(1,72) = 4.08, p = 0.047, η^2^ = 0.054), with younger participants having higher degrees. In other networks, neither clustering coefficient nor degree showed significant effects or interactions.

In the alpha band, the visual network exhibited significant interaction effects for the clustering coefficient (F(1,72) = 5.53, p = 0.021, η^2^ = 0.069). The post-hoc analysis revealed that older participants in the proactive driving condition had significantly lower clustering coefficients compared to younger participants (MD = −0.139, p = 0.039). The degree measure also showed a significant interaction effect in the visual network (F(1,72) = 4.49, p = 0.038, η^2^ = 0.055), indicating a similar trend where age and driving condition jointly influenced the network. The DMN showed significant driving condition effects for the degree measure (F(1,72) = 8.68, p = 0.004, η^2^ = 0.107), with reactive driving showing higher values. There were no significant age effects (F(1,72) = 0.007, p = 0.934, η^2^ = 0.0001) or interaction effects (F(1,72) = 0.62, p = 0.433, η^2^ = 0.008). The post-hoc analysis did not reveal significant pairwise differences.

## 4 Discussion

This study provides new insights into the differential impact of age on eye blink behavior, blink-related potentials and functional connectivity patterns. As a result, age effects were significant for blink duration, with younger adults having longer blink durations than older adults. The results also indicated age effects on ERP components, including the frontal N2, parietal N2, and occipital P0 and P2 components. The connectivity measures demonstrated age-related differences in brain network functionality, with older adults showing a reduction in clustering coefficients and degrees of various networks.

The observed shorter duration of blinks in older adults can be considered as a compensatory mechanism for maintaining visual attention and cognitive processing during driving which — due to age-related changes in sensory, motor, and cognitive functions — typically becomes more demanding with age [4,7]. In line with this assumption, previous research demonstrated that older adults blink with shorter durations when performing complex visual tasks for a longer time, possibly to maintain higher levels of wakefulness and attention [37]. Furthermore, the average duration of spontaneous blinks in younger subjects was found to be longer than that in older subjects, thus supporting the evidence that older adults blink with reduced duration as a compensatory mechanism [38].

According to the EEG results, aging affects neural responses during different driving conditions (reactive and proactive), exhibiting interesting trends in various bERP components. Specifically, the frontal N2 was reduced in the older group, which is in line with previous research demonstrating age effects on the N2 component [[39], [40], [41]]. The N2 is typically associated with cognitive control in demanding and conflictual situations when inhibitory control is needed [42,43]. These functions are organized in prefrontal cortex (PFC) networks [44], and age-related changes in these networks are often observed [45]. Also, Daffner et al. [46] showed age-related decreases in the anterior N2 component in response to new stimuli, suggesting that the ability of older people to process ambiguous representations is reduced [46]. Additionally, the occipital P2 component exhibits a similar effect to N2, which is manifested as a reduction in the availability of mental resources as tasks become more difficult [16].

The higher parietal N2 amplitude in older adults during reactive driving could reflect a greater amount of cognitive effort and resources needed by older adults to handle an unexpected driving challenge. For example, Karthaus et al. [24] discovered that older drivers use different driving strategies, either proactive-alert or reactive (indicated by low or high driving lane variability, respectively), and that these driving strategies differ significantly in terms of cognitive load and neural responses. More specifically, older adults using a reactive strategy had higher values of mental workload and increased values of frontal theta power. Besides, Getzmann et al. [13] revealed increased theta power in older drivers, suggesting more mental effort and cognitive control are needed to maintain performance. It should be noted that the two age groups did not differ in their performance in the lane-keeping task. Evidently, older adults are required to spend more cognitive resources on unexpected driving situations to achieve the same driving performance. Considering that the capability to respond to an unexpected challenge is critical for performance and safety, this effect is especially important for explaining age-related driving performance. Furthermore, the remarkable effect of age on the occipital P0 after blinks indicates that younger participants demonstrated a greater P0 amplitude, while older participants exhibited significantly lower amplitudes when driving reactively. This is consistent with the results of the study by Armstrong et al. [47]. The authors note that age might cause a degeneration of the level of neural efficiency and processing speed in humans. This was manifested by the late latencies of visual evoked potentials and the decrease in the amplitudes of the initial positive components of the occipital cortex with increasing age. These findings suggest that younger adults have more efficient early visual processing capabilities, which may contribute to better performance in tasks that require rapid visual responses.

The results of connectivity also reveal age-related differences in the efficacy of the brain network function at various frequencies. Consistent with prior research, the current study found that younger adults exhibited enhanced connectivity within the visual network compared to older adults, as evidenced by higher clustering coefficient and degree measures in the beta frequency band [48,49]. This is in line with other studies demonstrating that younger individuals have more efficient visual processing and attentional control during complex tasks like driving, which may be facilitated by their stronger visual network connectivity [48,50]. Similarly, the DMN showed age-related differences in the local efficiency of its network, with younger participants showing higher clustering coefficient values. This is consistent with research linking effective DMN modulation to cognitive control and task performance, and showing that aging is associated with decreased default mode flexibility during driving [48]. Related to these findings, the control network also had significant age effects, and the younger adult group showed higher clustering within this network. Geerligs et al. [51] reported that the deterioration of executive control networks in older adults may relate to declines in abilities of cognitive control and decision-making [51]. Another interesting finding related to age was a greater degree of connectivity in the limbic network under reactive conditions for the younger adult group. This suggests that younger adults may have better emotional regulation and stress response to unexpected events during driving.

On the other hand, the dorsal attention network in the theta frequency band showed an increase in both degree and clustering coefficient within the network in older adults, contrary to the results in other networks. This could potentially reflect a compensatory mechanism, where older adults attempt to recruit additional attentional resources to maintain cognitive performance, with such results being valuable due to prior reports on age-related dynamics in the attention network [11,12,52]. In line with this, some previous work has shown increased theta activity over frontal brain areas in old age [53,54], with increased fronto-central theta activity being associated with better performance in tasks requiring cognitive control [55,56]. These findings were discussed in the context of the compensation-related utilization of neural circuits (CRUNCH) hypothesis, which proposes that additional brain regions are activated in older age to compensate for declining brain function during cognitive tasks (for review, Kang et al. [57]). Additionally, the SM and SVA networks demonstrated significant effects of driving condition and age, respectively, on the degree of within the network, which could signify their involvement in the support of driving-related cognitive processes and their susceptibility to age-related changes. In the alpha band, the visual network indicated significant interaction effects between age and driving condition for clustering coefficient and degree, indicating that older adults in the proactive driving condition had lower connectivity, potentially contributing to their difficulty with anticipatory driving. In contrast, the DMN showed significant main effects of driving condition on degree, with the reactive condition associated with higher connectivity, likely due to the generalized age-related decline in DMN connectivity irrespective of task demands.

Despite these findings, several limitations should be considered. First, it is important to acknowledge that the interpretation of the P0 results requires caution. Specifically, although the higher P0 in the younger adults indicates better visual processing, other possible explanations should also be considered. Additionally, the P0 amplitude can also be influenced by factors such as head and eye movements. On the other hand, the scope of the P0 amplitude may also be affected by the head and eye movements. Therefore, the studies to be conducted in the future may employ the measures of eye movements in addition to using the EEG in studies. Second, while a stationary driving simulator is valuable for controlled experiments, it may not fully encompass the intricacies of real-life driving. To improve ecological validity, future studies could consider including on-road driving evaluations. Moreover, the sample size, particularly for the proactive driving group, was relatively small, which may limit the generalizability of the findings. Future studies should increase the sample size and include a broader range of demographics, which may provide a more complete picture of individual differences in driving performance and the corresponding neural activity differences during aging. Additionally, a within-subject manipulation of driving conditions could help to reduce variance unrelated to this factor. Finally, studies may look into additional neurophysiological indices and multimodal integration to gain a more complete picture of the cognitive and neural substrates that underpin driving across age groups.

## 5 Conclusion

The present study explores age-related differences in eye-blink behavior, blink-related potentials, and brain network connectivity during reactive and proactive driving tasks. Our findings show that younger adults blink longer than older adults, implying a compensatory mechanism for maintaining visual attention and cognitive processing. Additionally, there are notable age-related differences in frontal N2, parietal N2, and occipital P0 and P2 components which point out an increased cognitive load as well as altered neural processing among older people. Moreover, connectivity analyses show decreased clustering coefficients and degree measures in various networks especially those of visual and default mode networks, thus suggesting a reduced local efficiency along with integration among older people. In contrast, in older adults, the dorsal attention network has increased connectivity, suggesting a compensatory response to increased cognitive demands. This study highlights the complex interplay between aging, cognitive load, and neural connectivity, suggesting that older adults must exert more cognitive effort to maintain driving performance. Despite these insights, future studies could enhance ecological validity by including on-road driving evaluations and addressing sample diversity to capture broader demographic trends in neural responses during driving. Integrating eye movement measures and exploring multimodal neurophysiological indices could further illuminate the cognitive processes underlying age-related differences in driving performance.

## CRediT authorship contribution statement

**Emad Alyan:** Writing – original draft, Visualization, Methodology, Investigation, Formal analysis, Data curation, Conceptualization. **Stefan Arnau:** Writing – review & editing, Validation. **Stephan Getzmann:** Writing – review & editing, Validation, Resources. **Julian Elias Reiser:** Writing – review & editing, Validation. **Melanie Karthaus:** Writing – review & editing, Resources. **Edmund Wascher:** Writing – review & editing, Validation, Resources, Conceptualization.

## Data availability statement

The datasets are available from the corresponding author on reasonable request.

## Declaration of competing interest

The authors declare that they have no known competing financial interests or personal relationships that could have appeared to influence the work reported in this article.
