# Supplementary material for: Age-related differences in eye blink-related neural activity and functional connectivity during driving
Source: Heliyon. 2024 Dec 14;11(1):e41164. doi: 10.1016/j.heliyon.2024.e41164 (PMC11699334; doi:10.1016/j.heliyon.2024.e41164)
Supplement: Multimedia component 1 [file mmc1.docx]

**Age-related differences in eye blink-related neural activity and functional connectivity during driving: Supplementary material**

Emad Alyan, Stefan Arnau, Stephan Getzmann, Julian Elias Reiser, Melanie Karthaus, Edmund Wascher

**Clustering coefficients:**

| EEG band | Network | Source | Mean squares | F(1,72) | p-value | ƞ² | Post-hoc: Old vs. Young | | |
| --- | --- | --- | --- | --- | --- | --- | --- | --- | --- |
|  |  |  |  |  |  |  | Driving | MD | P-value |
| Theta | Vis | Age | 0.0179813 | 0.7036064 | 0.4043508 | 0.0096049 | Reactive | -0.0131966 | 0.9924556 |
|  |  | Driving | 0.0081355 | 0.3183423 | 0.574359 | 0.0043457 | Proactive | -0.0508001 | 0.8297801 |
|  |  | Interaction | 0.0062081 | 0.2429235 | 0.6236024 | 0.0033162 |  |  |  |
|  | SM | Age | 0.0002854 | 0.084339 | 0.7723361 | 0.0011538 | Reactive | 0.0065745 | 0.9811375 |
|  |  | Driving | 0.0028227 | 0.8341352 | 0.3641278 | 0.0114111 | Proactive | 0.0014881 | 0.9998835 |
|  |  | Interaction | 0.0001136 | 0.0335659 | 0.8551481 | 0.0004592 |  |  |  |
|  | DA | Age | 0.049971 | 6.934566 | 0.0103412 | 0.0878224 | Reactive | 0.0384404 | 0.4289661 |
|  |  | Driving | 0.0006048 | 0.0839276 | 0.7728764 | 0.0010629 | Proactive | 0.0682453 | 0.1464804 |
|  |  | Interaction | 0.0039001 | 0.5412288 | 0.4643138 | 0.0068544 |  |  |  |
|  | SVA | Age | 0.0018182 | 0.8460663 | 0.360739 | 0.0115496 | Reactive | -0.0012531 | 0.9997279 |
|  |  | Driving | 0.0001562 | 0.0727017 | 0.7882142 | 0.0009924 | Proactive | -0.0190972 | 0.6886922 |
|  |  | Interaction | 0.001398 | 0.6505029 | 0.4225891 | 0.00888 |  |  |  |
|  | Limbic | Age |  |  |  |  | Reactive |  |  |
|  |  | Driving |  |  |  |  | Proactive |  |  |
|  |  | Interaction |  |  |  |  |  |  |  |
|  | CON | Age | 0.0020011 | 0.8199388 | 0.3682183 | 0.0110976 | Reactive | -0.0186042 | 0.5867252 |
|  |  | Driving | 0.0010783 | 0.441815 | 0.5083711 | 0.0059798 | Proactive | -0.0027449 | 0.9988145 |
|  |  | Interaction | 0.0011043 | 0.4524701 | 0.5033174 | 0.006124 |  |  |  |
|  | DMN | Age | 0.0050985 | 0.8807844 | 0.3511246 | 0.0118193 | Reactive | -0.0300213 | 0.548637 |
|  |  | Driving | 0.0060222 | 1.0403552 | 0.3111539 | 0.0139606 | Proactive | -0.0040563 | 0.998952 |
|  |  | Interaction | 0.0029599 | 0.511339 | 0.4768722 | 0.0068617 |  |  |  |
| Alpha | Vis | Age | 0.0682565 | 3.6596147 | 0.0597235 | 0.0453658 | Reactive | 0.0143041 | 0.9848683 |
|  |  | Driving | 0.0140123 | 0.7512785 | 0.3889512 | 0.0093131 | Proactive | -0.1389906 | 0.039354 |
|  |  | Interaction | 0.1031715 | 5.5316018 | 0.0214144 | 0.0685716 |  |  |  |
|  | SM | Age | 0.0045377 | 0.9927844 | 0.3223997 | 0.0127314 | Reactive | 0.0135562 | 0.9063667 |
|  |  | Driving | 0.0091778 | 2.0079869 | 0.1607855 | 0.0257502 | Proactive | -0.045705 | 0.2768591 |
|  |  | Interaction | 0.0154186 | 3.3733952 | 0.0703847 | 0.0432601 |  |  |  |
|  | DA | Age | 0.0012535 | 0.3334204 | 0.5654533 | 0.0043867 | Reactive | -0.0029643 | 0.9984544 |
|  |  | Driving | 0.0125723 | 3.3441893 | 0.0715843 | 0.0439984 | Proactive | 0.0198611 | 0.8215521 |
|  |  | Interaction | 0.0022874 | 0.6084374 | 0.4379335 | 0.008005 |  |  |  |
|  | SVA | Age | 0.000104 | 0.0269398 | 0.870086 | 0.0003646 | Reactive | -0.017831 | 0.7694898 |
|  |  | Driving | 1.519E-06 | 0.0003933 | 0.9842323 | 5.323E-06 | Proactive | 0.0226992 | 0.7622633 |
|  |  | Interaction | 0.0072121 | 1.8673503 | 0.1760313 | 0.0252724 |  |  |  |
|  | Limbic | Age | 0.0093524 | 2.026345 | 0.1589111 | 0.0263252 | Reactive | 6.939E-18 | 1 |
|  |  | Driving | 0.0093524 | 2.026345 | 0.1589111 | 0.0263252 | Proactive | 0.0461538 | 0.272735 |
|  |  | Interaction | 0.0093524 | 2.026345 | 0.1589111 | 0.0263252 |  |  |  |
|  | CON | Age | 0.0052838 | 2.6386006 | 0.1086655 | 0.035349 | Reactive | -0.0141556 | 0.7122176 |
|  |  | Driving | 0.000134 | 0.0669174 | 0.7966172 | 0.0008965 | Proactive | -0.0205357 | 0.6106368 |
|  |  | Interaction | 0.0001787 | 0.0892467 | 0.7659976 | 0.0011956 |  |  |  |
|  | DMN | Age | 0.0041508 | 0.6775605 | 0.4131461 | 0.0090063 | Reactive | -0.0168466 | 0.8871398 |
|  |  | Driving | 0.0002155 | 0.0351761 | 0.8517547 | 0.0004676 | Proactive | 0.0475944 | 0.36932 |
|  |  | Interaction | 0.0182318 | 2.9760906 | 0.0887933 | 0.0395589 |  |  |  |
| Beta | Vis | Age | 1.2603505 | 25.499101 | 3.219E-06 | 0.2570608 | Reactive | -0.2339097 | 0.0038594 |
|  |  | Driving | 0.0109851 | 0.2222473 | 0.6387583 | 0.0022405 | Proactive | -0.3018782 | 0.0028425 |
|  |  | Interaction | 0.0202824 | 0.4103494 | 0.5238258 | 0.0041368 |  |  |  |
|  | SM | Age | 0.025128 | 0.6439408 | 0.4249282 | 0.0088436 | Reactive | -0.0471231 | 0.8529971 |
|  |  | Driving | 0.0028263 | 0.0724267 | 0.7886054 | 0.0009947 | Proactive | -0.0285299 | 0.9801837 |
|  |  | Interaction | 0.0015178 | 0.0388956 | 0.8442113 | 0.0005342 |  |  |  |
|  | DA | Age | 0.0170038 | 0.3699929 | 0.5449222 | 0.0050982 | Reactive | -0.0140054 | 0.9962183 |
|  |  | Driving | 0.0102835 | 0.2237633 | 0.6376182 | 0.0030833 | Proactive | -0.0482275 | 0.930955 |
|  |  | Interaction | 0.0051418 | 0.1118836 | 0.7389823 | 0.0015417 |  |  |  |
|  | SVA | Age | 0.0160403 | 0.4269486 | 0.5155713 | 0.0057681 | Reactive | -0.0589302 | 0.736636 |
|  |  | Driving | 0.0453977 | 1.2083622 | 0.2753188 | 0.0163251 | Proactive | -0.0015139 | 0.9999967 |
|  |  | Interaction | 0.0144736 | 0.3852473 | 0.5367681 | 0.0052047 |  |  |  |
|  | Limbic | Age | 0.0333683 | 0.8569108 | 0.3576971 | 0.0110425 | Reactive | -0.1083333 | 0.2602054 |
|  |  | Driving | 0.1039151 | 2.6685802 | 0.1067129 | 0.0343883 | Proactive | 0.0211538 | 0.9916837 |
|  |  | Interaction | 0.0736138 | 1.8904297 | 0.1734171 | 0.0243608 |  |  |  |
|  | CON | Age | 0.2030846 | 4.0324223 | 0.0483862 | 0.0526796 | Reactive | -0.1190525 | 0.2889979 |
|  |  | Driving | 0.0136895 | 0.2718175 | 0.6037152 | 0.003551 | Proactive | -0.0960204 | 0.6625393 |
|  |  | Interaction | 0.002329 | 0.0462449 | 0.8303392 | 0.0006041 |  |  |  |
|  | DMN | Age | 0.2055307 | 5.9671262 | 0.0170296 | 0.0752677 | Reactive | -0.1251209 | 0.1151991 |
|  |  | Driving | 0.0346658 | 1.0064458 | 0.3191156 | 0.012695 | Proactive | -0.0912434 | 0.5554621 |
|  |  | Interaction | 0.0050388 | 0.1462907 | 0.7032324 | 0.0018453 |  |  |  |

**Degree:**

| EEG band | Network | Source | Mean squares | F(1,72) | p-value | ƞ² | Post-hoc: Old vs. Young | | |
| --- | --- | --- | --- | --- | --- | --- | --- | --- | --- |
|  |  |  |  |  |  |  | Driving | MD | P-value |
| Theta | Vis | Age | 5.321466564 | 2.378933651 | 0.127365451 | 0.031725918 | Reactive | -0.39562 | 0.810078 |
|  |  | Driving | 0.691832887 | 0.309280255 | 0.579846441 | 0.004124621 | Proactive | -0.70532 | 0.589264 |
|  |  | Interaction | 0.4210899 | 0.188246026 | 0.665677778 | 0.002510485 |  |  |  |
|  | SM | Age | 0.000714867 | 0.003377925 | 0.953814113 | 4.38664E-05 | Reactive | -0.09586 | 0.89632 |
|  |  | Driving | 0.925738105 | 4.374346276 | 0.040012113 | 0.056806102 | Proactive | 0.083104 | 0.962457 |
|  |  | Interaction | 0.140624631 | 0.664486888 | 0.41766869 | 0.008629154 |  |  |  |
|  | DA | Age | 3.497744489 | 8.728285763 | 0.004230849 | 0.108107275 | Reactive | 0.355388 | 0.241947 |
|  |  | Driving | 0.092243423 | 0.23018461 | 0.632841243 | 0.002851033 | Proactive | 0.537179 | 0.114118 |
|  |  | Interaction | 0.145094293 | 0.362068886 | 0.549249542 | 0.004484532 |  |  |  |
|  | SVA | Age | 0.972368314 | 4.084740295 | 0.046991076 | 0.053573365 | Reactive | -0.1485 | 0.736 |
|  |  | Driving | 6.12571E-07 | 2.5733E-06 | 0.99872451 | 3.37501E-08 | Proactive | -0.32212 | 0.29704 |
|  |  | Interaction | 0.132342922 | 0.555948252 | 0.458324703 | 0.007291533 |  |  |  |
|  | Limbic | Age | 0.190547356 | 2.226190316 | 0.140056896 | 0.029510921 | Reactive | -0.0391 | 0.969497 |
|  |  | Driving | 0.100352149 | 1.172427614 | 0.282515942 | 0.015541986 | Proactive | -0.16923 | 0.414045 |
|  |  | Interaction | 0.074349927 | 0.868640168 | 0.354446841 | 0.011514906 |  |  |  |
|  | CON | Age | 0.123078954 | 0.315643932 | 0.575982106 | 0.004280323 | Reactive | 0.05668 | 0.990033 |
|  |  | Driving | 0.305035608 | 0.782283531 | 0.379387761 | 0.010608239 | Proactive | -0.22411 | 0.771786 |
|  |  | Interaction | 0.346159915 | 0.887749475 | 0.349238649 | 0.012038421 |  |  |  |
|  | DMN | Age | 0.262905349 | 0.469272087 | 0.495521744 | 0.006292782 | Reactive | -0.28838 | 0.568321 |
|  |  | Driving | 0.634389593 | 1.132351735 | 0.290830079 | 0.015184459 | Proactive | 0.04367 | 0.998629 |
|  |  | Interaction | 0.484066232 | 0.864032518 | 0.355718709 | 0.011586388 |  |  |  |
| Alpha | Vis | Age | 4.853136027 | 3.562671552 | 0.063123113 | 0.043512132 | Reactive | 0.064573 | 0.997691 |
|  |  | Driving | 3.525497035 | 2.588056037 | 0.112048821 | 0.031608818 | Proactive | -1.11595 | 0.059208 |
|  |  | Interaction | 6.118637356 | 4.491672009 | 0.037509337 | 0.054858333 |  |  |  |
|  | SM | Age | 0.366360684 | 0.738275656 | 0.393065539 | 0.009788614 | Reactive | 0.130773 | 0.923864 |
|  |  | Driving | 0.20046541 | 0.403970018 | 0.527061091 | 0.005356138 | Proactive | -0.41964 | 0.387701 |
|  |  | Interaction | 1.330111174 | 2.680387778 | 0.105954729 | 0.035538596 |  |  |  |
|  | DA | Age | 0.039947398 | 0.149465846 | 0.700186574 | 0.002033739 | Reactive | -0.05013 | 0.987914 |
|  |  | Driving | 0.224919801 | 0.841552395 | 0.362015888 | 0.011450765 | Proactive | 0.145513 | 0.874747 |
|  |  | Interaction | 0.168039941 | 0.628732614 | 0.43042574 | 0.008554986 |  |  |  |
|  | SVA | Age | 0.011105495 | 0.038854266 | 0.844293047 | 0.000524714 | Reactive | -0.10996 | 0.899835 |
|  |  | Driving | 0.26856022 | 0.939598821 | 0.335626054 | 0.01268898 | Proactive | 0.160256 | 0.8529 |
|  |  | Interaction | 0.320580182 | 1.121598577 | 0.293114558 | 0.015146828 |  |  |  |
|  | Limbic | Age | 0.07626212 | 0.660951716 | 0.41890438 | 0.008741435 | Reactive | -0.17218 | 0.328449 |
|  |  | Driving | 0.103076911 | 0.893351266 | 0.347731913 | 0.011815042 | Proactive | 0.040385 | 0.988738 |
|  |  | Interaction | 0.19837607 | 1.719293985 | 0.193950312 | 0.02273857 |  |  |  |
|  | CON | Age | 0.042679302 | 0.129948666 | 0.719540689 | 0.001749183 | Reactive | -0.1122 | 0.912142 |
|  |  | Driving | 0.285909221 | 0.870527865 | 0.353927593 | 0.0117178 | Proactive | 0.210799 | 0.758361 |
|  |  | Interaction | 0.458054339 | 1.394670185 | 0.241504451 | 0.018773054 |  |  |  |
|  | DMN | Age | 0.002930784 | 0.006945877 | 0.93381094 | 8.53765E-05 | Reactive | -0.10918 | 0.941973 |
|  |  | Driving | 3.660925988 | 8.676292686 | 0.004339704 | 0.106646226 | Proactive | 0.135016 | 0.944439 |
|  |  | Interaction | 0.261806139 | 0.620473262 | 0.433457035 | 0.00762666 |  |  |  |
| Beta | Vis | Age | 255.0171823 | 31.14207312 | 3.97578E-07 | 0.298112869 | Reactive | -3.21694 | 0.001775 |
|  |  | Driving | 0.664884683 | 0.081194087 | 0.776503722 | 0.000777244 | Proactive | -4.40441 | 0.000565 |
|  |  | Interaction | 6.190881819 | 0.756015311 | 0.387467816 | 0.007237087 |  |  |  |
|  | SM | Age | 0.565244815 | 0.159955282 | 0.690381775 | 0.002209594 | Reactive | -0.0051 | 1 |
|  |  | Driving | 0.589614124 | 0.166851409 | 0.684137682 | 0.002304856 | Proactive | -0.35371 | 0.957897 |
|  |  | Interaction | 0.533552404 | 0.150986835 | 0.698740746 | 0.002085705 |  |  |  |
|  | DA | Age | 2.215137319 | 0.597345808 | 0.442122421 | 0.008203601 | Reactive | -0.06992 | 0.999342 |
|  |  | Driving | 0.106226212 | 0.02864553 | 0.86607469 | 0.000393401 | Proactive | -0.64038 | 0.809733 |
|  |  | Interaction | 1.428747857 | 0.385283809 | 0.536748889 | 0.005291264 |  |  |  |
|  | SVA | Age | 0.333968608 | 0.143469818 | 0.705970275 | 0.001979828 | Reactive | -0.26378 | 0.937285 |
|  |  | Driving | 0.417958804 | 0.179551227 | 0.673022423 | 0.002477737 | Proactive | -0.01202 | 0.999997 |
|  |  | Interaction | 0.278289616 | 0.119550639 | 0.730530614 | 0.001649753 |  |  |  |
|  | Limbic | Age | 2.730296853 | 5.074364338 | 0.027330799 | 0.061397103 | Reactive | -0.64436 | 0.021464 |
|  |  | Driving | 1.484323198 | 2.75867318 | 0.101077372 | 0.033378475 | Proactive | -0.14423 | 0.952379 |
|  |  | Interaction | 1.098174967 | 2.04100147 | 0.157432792 | 0.02469503 |  |  |  |
|  | CON | Age | 9.732550944 | 2.806963399 | 0.098193938 | 0.037335933 | Reactive | -0.8343 | 0.438445 |
|  |  | Driving | 0.568246226 | 0.1638878 | 0.686802292 | 0.002179902 | Proactive | -0.65459 | 0.782649 |
|  |  | Interaction | 0.141793771 | 0.040894718 | 0.840311599 | 0.000543948 |  |  |  |
|  | DMN | Age | 26.56536873 | 2.844022326 | 0.096043203 | 0.036936232 | Reactive | -1.70943 | 0.24495 |
|  |  | Driving | 15.28854759 | 1.636753893 | 0.204879014 | 0.021257049 | Proactive | -0.7504 | 0.91255 |
|  |  | Interaction | 4.038026293 | 0.432301055 | 0.512958531 | 0.005614433 |  |  |  |
